# Investigating the plant growth promoting and biocontrol potentiality of endophytic *Streptomyces* SP. SP5 against early blight in *Solanum lycopersicum* seedlings

**DOI:** 10.1186/s12866-022-02695-8

**Published:** 2022-11-29

**Authors:** Sapna Devi, Manish Sharma, Rajesh Kumari Manhas

**Affiliations:** Department of Microbiology, Guru Nanak Dev University, Amritsar, Punjab India

**Keywords:** *Streptomyces* SP. SP5, Plant growth promotion, Biocontrol, Indole acetic acid

## Abstract

**Background:**

Early blight (EB), caused by *Alternaria solani*, is one of the alarming diseases that restrict tomato production globally. Existing cultural practices and fungicide applications are not enough to control early blight diseases. Therefore, the study aimed to isolate, identify, and characterize an endophytic *Streptomyces* exhibiting the potential to control early blight in tomato and also promote plant growth.

**Results:**

From a *Citrus jambhiri* leaf, an endophytic *Streptomyces* sp. with antagonistic activity against *Alternaria solani, Colletotrichum acutatum*, *Cladosporium herbarum*, *Alternaria brassicicola*, *Alternaria* sp., *Fusarium oxysporum* and *Fusarium* sp. was isolated*.* It was identified as a *Streptomyces* sp. through 16S ribosomal DNA sequence analysis and designated as SP5. It also produced indole acetic acid which was confirmed by Salkowski reagent assay, TLC and HPLC analysis. Treatment of pathogen infected plants with *Streptomyces* sp. SP5 antagonists (culture cells/culture supernatant/solvent extract/ acetone precipitates) decreased the early blight disease incidence and significantly increased the various agronomic traits.

**Conclusion:**

The present study concluded that *Streptomyces* sp. SP5 possessed antifungal activity against different fungal phytopathogens and had significant potential to control early blight disease and promote plant growth.

**Supplementary Information:**

The online version contains supplementary material available at 10.1186/s12866-022-02695-8.

## Background

Tomato is the second most widely used vegetable in the world after potato because of its high nutritional value (rich in vitamins A and C) and the presence of lycopene and beta-carotene pigments [1, 2]. Tomato crop occupies an area of 4.85 million hectares in the world with the production of 182.3 million tons and the productivity of 37.6 t/ha [3]. In India, the tomato crop is grown in an area of 0.5 million ha with an annual production of 7.4 million tons [4]. Continuous cropping of tomato plants leads to the prevalence of fungal diseases. One of the most devastating diseases of tomato is early blight caused by the necrotrophic fungus *Alternaria solani* [5]. Leaf blight, stem blight, and apical fruit rot are the most damaging symptoms of the disease, and in severe cases, this can lead to complete defoliation [6]. Many reports recorded that early blight is responsible for a large proportion of total monetary loss sustained by tomato producers every growing season [7]. Pesticide application is still a valuable and efficient method to controlling plant diseases in modern agriculture. However, the use of agrochemicals is falling into disfavor because of the accretion of toxic compounds potentially perilous to humans and the environment. Also, in the increase of resistance of the pathogens to agrochemicals and effects on a variety of non-target organisms, they are not considered to be a long-term solution. Therefore, recent efforts have been focused on developing eco-friendly safe, long-lasting, and effective agents against plant pathogens for the management of plant diseases. Endophytic microorganisms have become the center of intensive research because they provide numerous advantages over chemical fertilizers [8]. These include environmental protection, safety for humans, reduction of chemical pesticide residues in food, and increased biodiversity in a managed ecosystem. Many recent studies have discussed the possible use of microbe-based biocontrol agents as agrochemical substitutes or supplements [9, 10]. Various bacteria and fungi, such as *Streptomyces*, *Bacillus*, *Pseudomonas*, and *Trichoderma* as well as nonpathogenic *Fusarium*, have been successfully used as biocontrol agents [11, 12].

Various beneficial bacteria support their host plants by increasing growth and/or by protecting them against pathogenic diseases [13]. Among all bacterial communities, actinobacteria, especially *Streptomyces* have been reported to play an important role in the plant rhizosphere by secreting a wide range of antimicrobial products. *Streptomyces* characterized by a wide range of modes of action like antibiotic production, lysis of fungal cell walls, competition, and hyper parasitism have been proved to be effective biocontrol agents [14]. Besides acting as biocontrol agents, they also possess the potential to stimulate plant growth either directly or indirectly [15]. *Streptomyces* are also known to develop symbiotic associations with crop plants, colonizing their internal tissues without causing disease symptoms and producing phytohormones such as gibberellic acid and indole-3-acetic acid (IAA) [16, 17]. Indole acetic acid (IAA), one of the most important phytohormones, has a vital role in the stimulation of root growth. The production of IAA has been reported in various *Streptomyces* species [18, 19]. Commercial formulations from *Streptomyces* have already been successfully developed to control fungal phytopathogens. Mycostop, a *Streptomyces*- based (*Streptomyces griseoviridis* K61) biocontrol product, is registered for use in Canada and 15 other countries against *Fusarium, Alternaria* and *Phytophthora.* Another product, Actinovate (*Streptomyces lydicus* WYEC108) is recommended for foliar and soil-borne fungal diseases of greenhouse and field-grown crops [20].

The natural habitat of most *Streptomyces* is the soil where they may constitute from 1 to 20% of the culturable population, but *Streptomyces* are also present inside plant tissues as endophytes [21–23]. To discover new biocontrol agents there is a need to screen new *Streptomyces* strains from untapped sources. Therefore, in the present study an endophytic isolate SP5, exhibiting antifungal and plant growth-promoting activities, was recovered from *Citrus jambhiri* leaves. The aim of this study was to isolate a potent endophytic *Streptomyces* strain having potential to control early blight disease in *S. lycopersicum* plants caused by *A. solani.*

During this investigation the ability of the strain to promote plant growth in vivo was also assessed by observing its impact on various agronomic traits in the *S. lycopersicum* plant.

## Results

### Isolation and screening

A total of 42 different endophytic actinobacteria isolates were recovered from plant samples. Among these, 15 isolates demonstrated antifungal activity against one or more test fungi during primary screening, and 9 isolates were found to have antifungal activity in the culture supernatant, inhibiting various test fungi to varying degrees (Table S1). Isolate SP5 was selected for further studies because it exhibited potent antifungal activity against all the tested phytopathogenic fungi.

### Phenotypic and phylogenetic analysis of *Streptomyces* SP. SP5

**Fig. 1:**
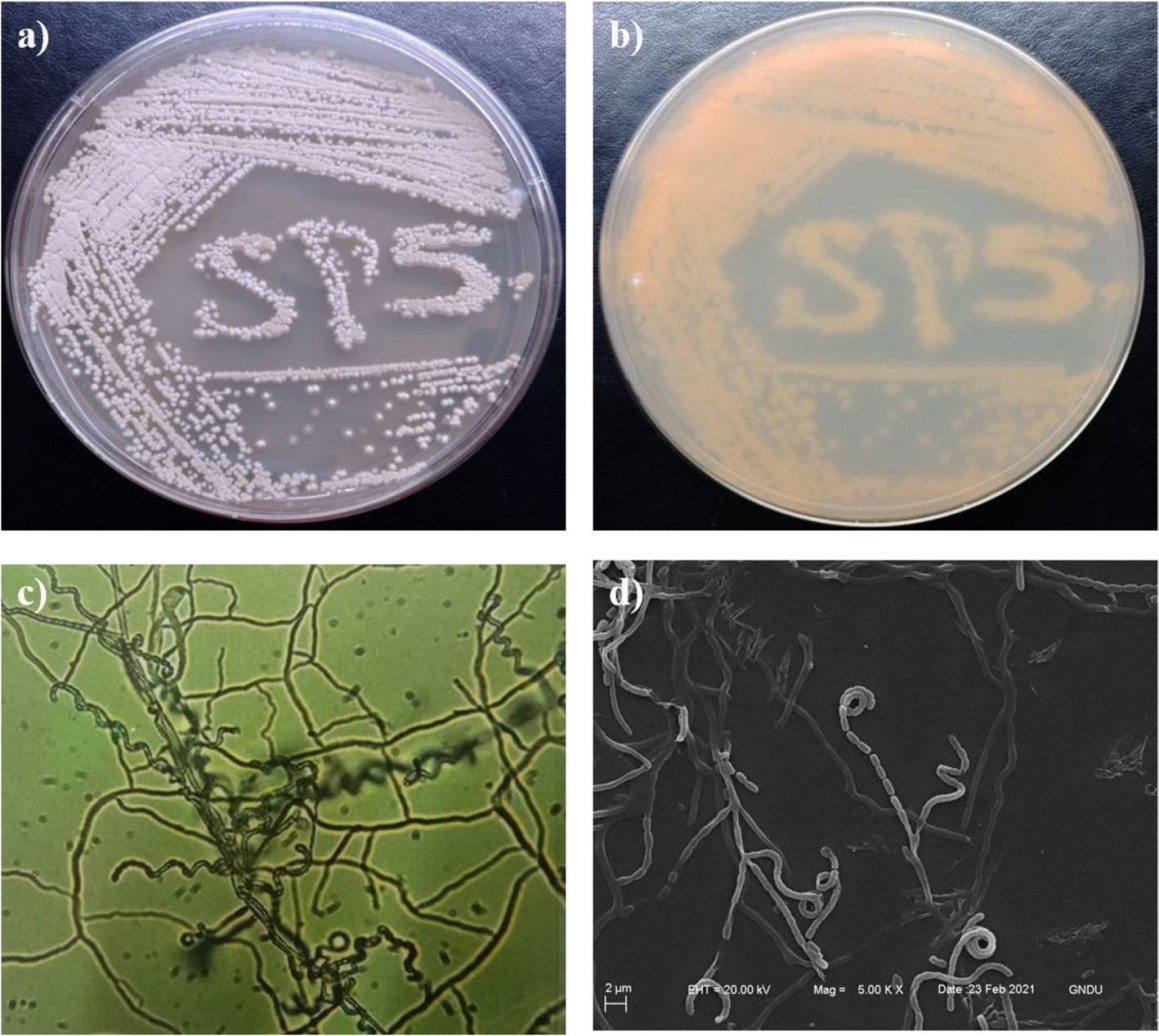
*Streptomyces* sp. SP5 morphological characteristics: **a** Aerial mycelium, **b** Substratum mycelium, **c** Aerial hyphae spore chains with open hooks under Light Microscope (100X), **d** Electron microscopic image of spores with smooth surface (Magnification = 5.00 KX

**Fig. 2:**
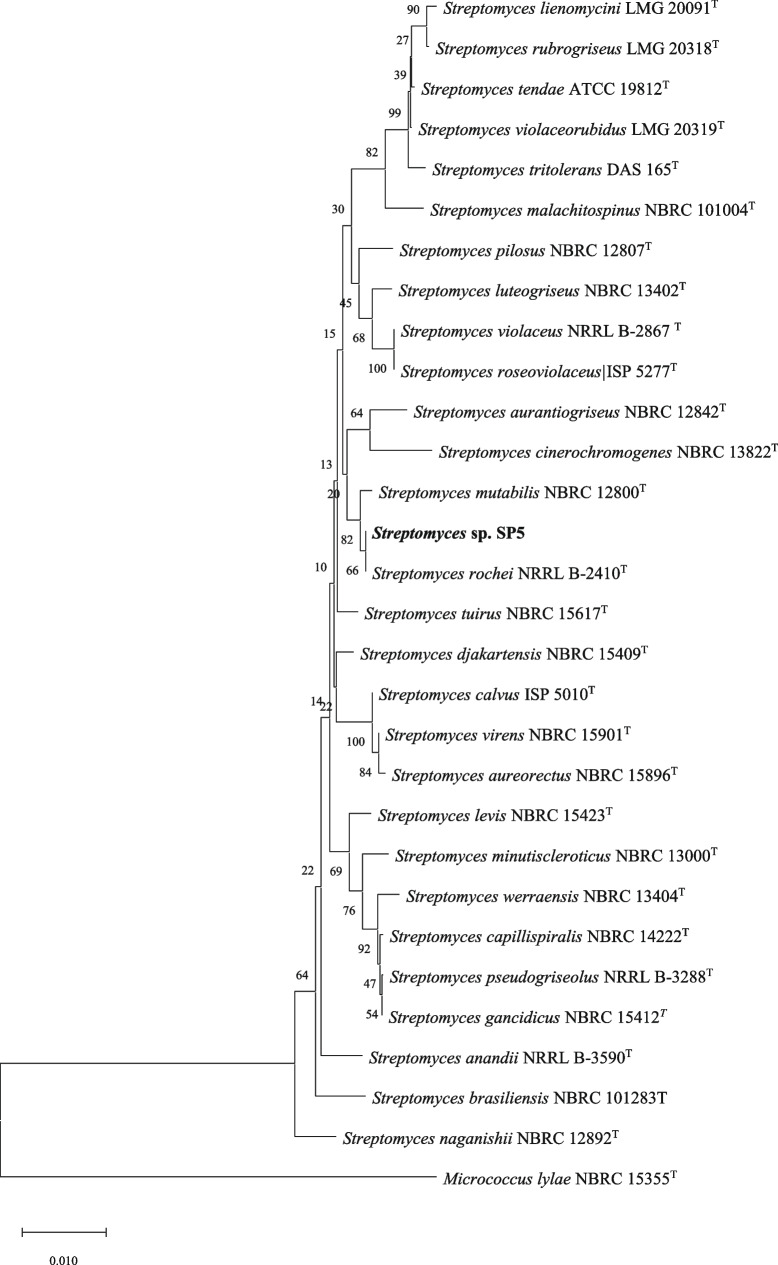
Phylogenetic tree obtained by neighbour joining analysis of 16S rRNA gene sequences showing the relationship between SP5 and related species belonging to genus *Streptomyces* obtained from EzTaxon database. Numbers on branch nodes are bootstrap values (expressed as percentage of 1000 replications)

*Streptomyces* sp. SP5 showed good growth on SCNA (Starch casein nitrate agar) and all ISP (International *Streptomyces* project) media except the ISP2 medium. It exhibited varied cultural characteristics on different media (Table S2). Pigmentation was not observed in any ISP medium. On SCNA medium isolate showed yellowish-white sporulation (Fig. 1a) with strong yellow aerial and brilliant yellow substrate mycelia (Fig. 1b). Under the light microscope (100X), flexuous spore chains with hooks were observed (Fig. 1c), and it was assigned to the *Streptomyces* retinaculiaperti group. SEM micromorphological analysis showed spore chains, each bearing 20–30 cylindrical spores (1.5–2.0 m length and 1.5 m width) with smooth surface (Fig. 1d) on aerial mycelium. The chemotaxonomic analysis revealed the presence of LL-DAP (LL-diaminopimelic acid) in the cell wall, and no characteristic sugar in whole-cell hydrolysate. In physiological studies, *Streptomyces* sp. SP5 grew at temperature of 25–45 °C (optimum at 28 °C), pH of 5–10 (optimum at pH 7.0) and showed salt tolerance up to 5%. In biochemical characteristics, isolate SP5 hydrolyzed starch, cellulose, lipid, gelatin and esculin but not able to hydrolyze casein. Isolate SP5 showed positive results for the production of catalase, oxidase, hydrogen sulphide and citrase but gave negative results for both indole production and MRVP (Methyl Red–Voges-Proskauer) tests. The isolate was able to utilize all the tested sugars as the sole carbon source except arabinose (Additional file Table S3). These cultural and chemotaxonomic characteristics showed that SP5 belongs to the genus *Streptomyces* and was further confirmed by 16S rRNA sequencing. Almost complete 16SrRNA gene sequence (1400 bp) of *Streptomyces* sp. SP5 was determined and aligned, using the EzTaxon database. The 16S rRNA gene sequence has been deposited in the GenBank database with accession number MW564023. Nucleotide BLAST search analysis showed 100% pairwise similarity with *Streptomyces rochei* NRRL B- 2410. *Streptomyces* sp. SP5 formed a clade with *Streptomyces rochei* NRRL B- 2410 in phylogenetic tree constructed using neighbor-joining method with bootstrap value of 64% (Fig. 2). The culture has been deposited in International Depository Authority (MTCC and Gene Bank, CSIR-IMTECH, Chandigarh (India) with accession number MTCC-13071.

### Antifungal potential

**Fig. 3:**
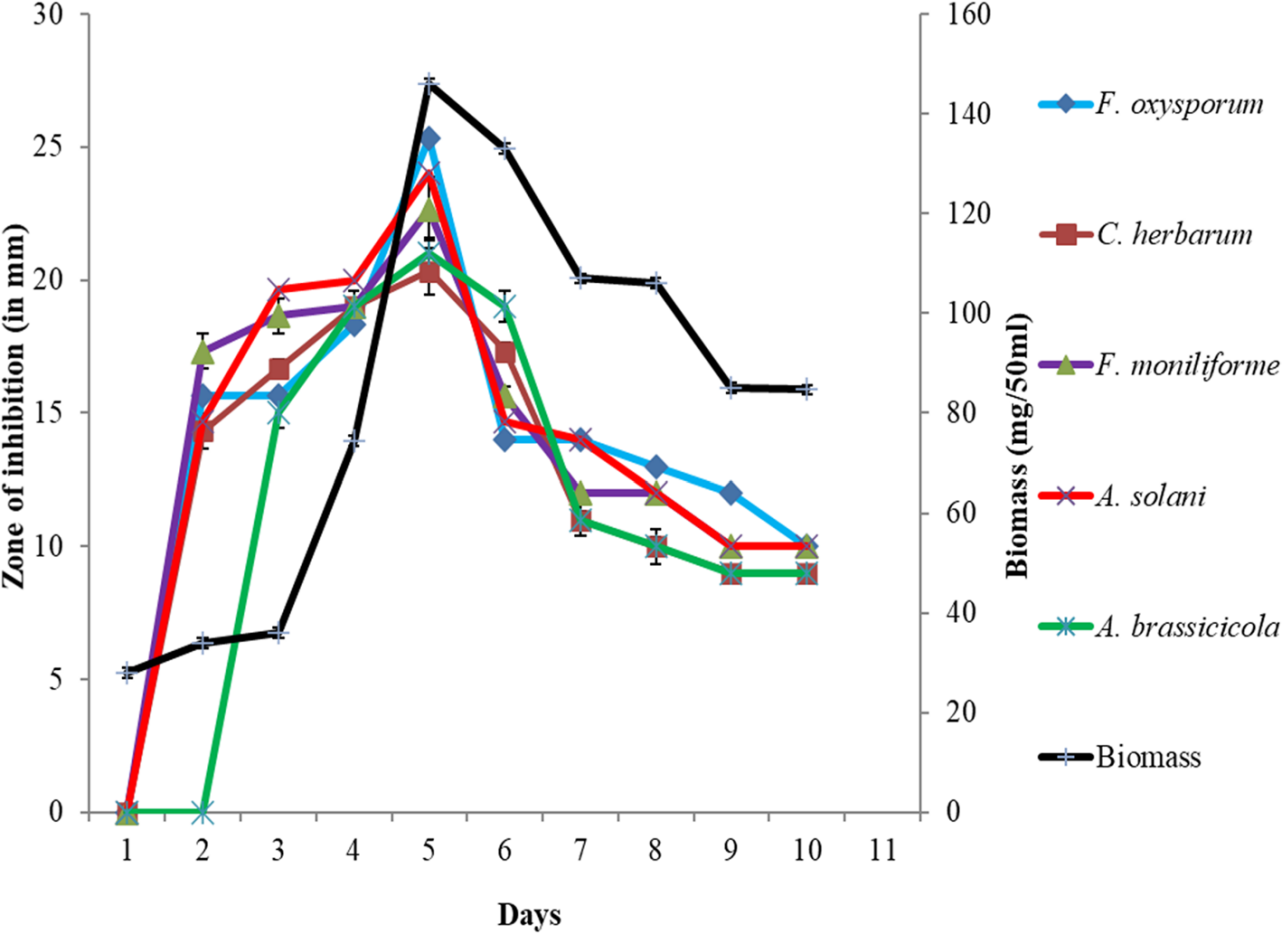
Growth and antifungal activity of *Streptomyces* sp. SP5 against test organisms viz. *Fusarium oxysporum*, *Colletotrichum herbarum*, *Fusarium* sp. *Alternaria solani* and *Alternaria* sp

**Fig. 4:**
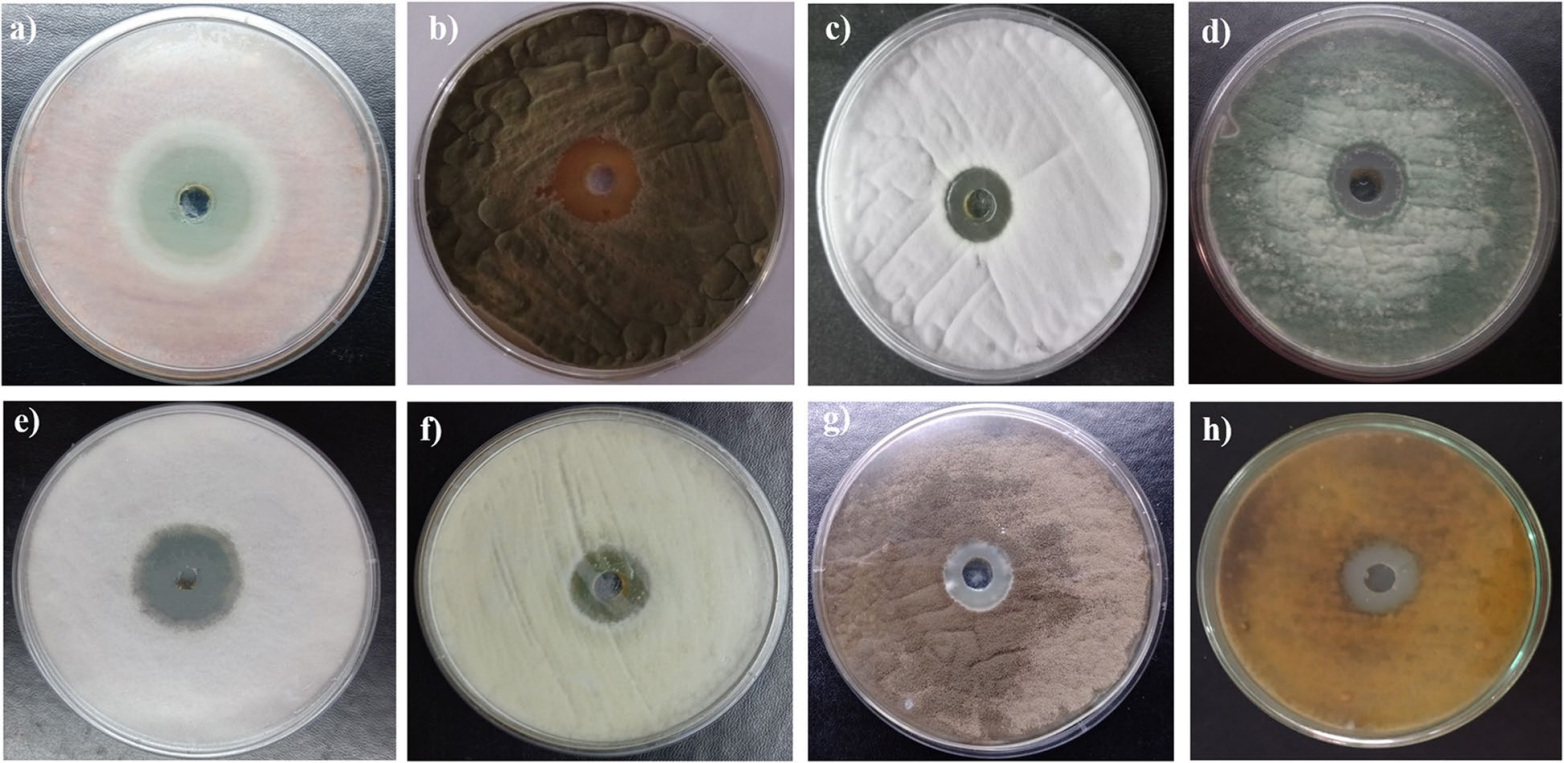
Antifungal activity of *Streptomyces* sp. SP5 cultural supernatant against fungal phytopathogens: **a) ***Fusarium* sp.; **b) ***Cladosporium herbarum; ***c) ***Colletotrichum acutatum; ***d) ***Alternaria* sp.; **e) ***Fusarium oxysporum; ***f) ***Fusarium solani; ***g) ***Alternaria solani; ***h) ***Alternaria alternata* (Production carried out in SCN broth, pH 7.0 at 28 °C using 2.5% inoculum)

*Streptomyces* sp. SP5 showed antifungal activity against all the tested fungal phytopathogens. The production of active metabolites in SCN culture broth against fungal pathogens was detected after 2 days of incubation. The maximum activity reached after 5 days, and then began to decrease as the incubation period was extended further. Additionally, during the 10-day activity profile, there was a positive correlation between antifungal activity and biomass (Fig. 3, Table S4). The maximum biomass was obtained after fifth day of incubation, which coincided with the maximum activity obtained on the same day. In vitro bioassay demonstrated broad-spectrum antifungal activity of *Streptomyces* sp. SP5 against test fungi (Fig. 4). Among the tested fungi, higher activity was observed against *Fusarium oxysporum*, *Cladosporium herbarum*, *Fusarium* sp. (25-28 mm) as compared to *Alternaria brassicicola*, *Alternaria* sp.*, Colletotrichum acutatum, Alternaria solani,* and *Fusarium solani* (20-25 mm) (Table S5).

### Antifungal metabolite extraction

The active metabolites from culture supernatant were extracted in diethyl ether. After the concentration of the organic phase using rotavapor, a light-yellow colored crude extract was obtained, which was redissolved in chloroform. Diethyl ether solvent extract showed pronounced activity against *A. brassicicola*, *A. solani*, *F. oxysporum* and *F. solani* (15-20 mm). However, the activity was also observed in the aqueous phase which indicated that antifungal compounds were not fully recovered by solvent extraction. Therefore, another technique i.e., acetone precipitation was employed to recover the bioactive metabolites from the culture supernatant. The acetone precipitates showed antifungal activity against *A. brassicicola*, *A. solani*, *C. acutatum, F. oxysporum*, *F. solani* and *C. herbarum* (20-30 mm).

### Stability of antifungal metabolites in the culture supernatant

The stability of antifungal metabolites present in the culture supernatant was investigated under various physical stresses. Active metabolites remained completely stable at 37 °C after 1 h exposure. However, a loss of 20, 48, and 68% in the activity was observed at 70 °C, 100 °C, and autoclaving (121 °C for 20 min), respectively. The metabolites remained active at extreme pH values (from 2 to 14) with 92 and 88% residual activity, respectively. The metabolites were photostable in both UV and sunlight (Table 1).

**Table 1:** Effect of various parameters on antifungal activity of culture supernatant against *A. solani*

Treatment	*Streptomyces* sp. SP5	
	Zone of inhibition (mm) against *A. solani*	% Residual activity
**Control (Untreated)**	25 ± 0.0	100
**Temperature treatment**		
37 °C, 1 h	25.0 ± 0.2	100
50 °C, 1 h	23.0 ± 0.4	92
70 °C, 1 h	20.0 ± 1.0	80
100 °C, 1 h	13.0 ± 0.3	52
121 °C, 45 min	8.0 ± 0.1	32
-20 °C, 1 h	25.0 ± 0.0	100
**pH tolerance**		
pH 2	22.0 ± 0.3	88
pH 14	23.0 ± 0.1	92
**Photostability**		
Sunlight, 1 h	25.0 ± 0.3	100
UV light, 1 h	25.0 ± 0.1	100

Values represented as mean ± SE (*n* = 3)

### IAA production from *Streptomyces* SP. SP5

**Fig. 5:**
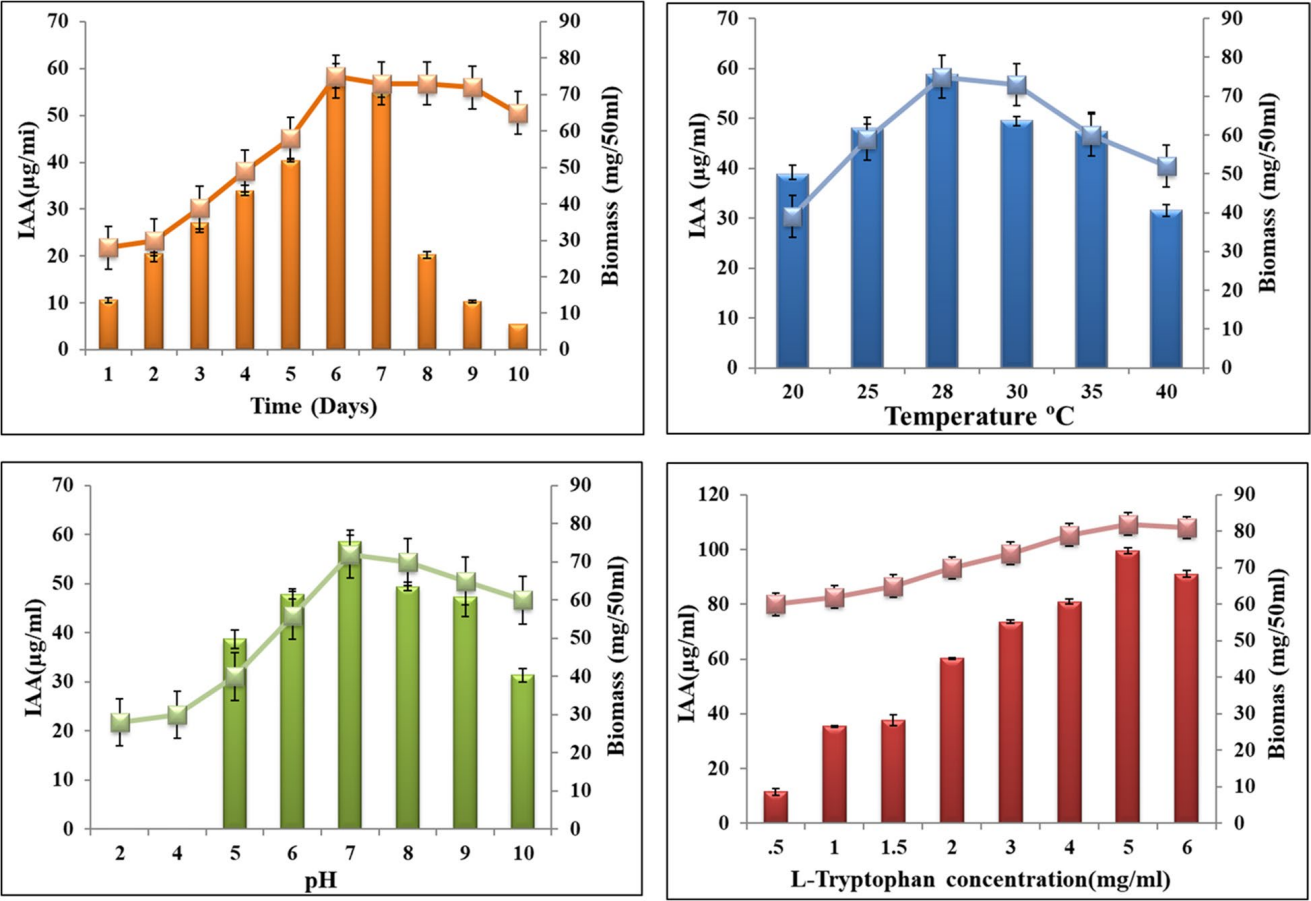
*Streptomyces* sp. SP5 produces IAA in response to **a)** incubation period, **b)** pH, **c)** temperature, and **d)** tryptophan concentration. Columns and bars represent the mean ± SE

*Streptomyces* sp. SP5 produced 40 μg/ml indole acetic acid in an initial screening for indole acetic acid production. TLC and HPLC analyses further demonstrated the ability of *Streptomyces* sp. SP5 to produce IAA. The ethyl acetate crude extract displayed a clear pink-colored spot on the TLC plate after being treated with Salkowski regent at Rf value corresponding to standard IAA (0.9 retention factor). After the detection of IAA in TLC, partial purification of crude extract containing IAA was done using silica gel chromatography. HPLC analysis was used to precisely classify and quantify the IAA production. The partially purified extract and corresponding reference authentic standard both displayed peaks at the same retention time (18.6 min) (Fig. S1). For the highest IAA production by *Streptomyces* sp. SP5, experiments were carried out with different values of the incubation period, temperature, pH, and L-tryptophan concentration. As shown in Fig. 5a, IAA production began within 24 hours of incubation, increased steadily over the time, reached a maximum of 58.43 μg/ml after 6 days, then began to decline with additional incubation, reaching 5.45 μg/ml after 10 days. The impact of pH on IAA production was investigated within the pH range of 5 to 10. *Streptomyces* sp. SP5 produced the maximum IAA of 58.63 μg/ml at an initial pH of 7.0. Acidity and high alkalinity of the medium led to a marked decrease in growth and IAA production (Fig. 5b). Incubation temperature had a significant impact on IAA production. The maximum production of 58.63 μg/ml IAA was achieved at 28 °C (Fig. 5c). In the absence of L- tryptophan i.e. IAA precursor, *Streptomyces* did not produce IAA. With the increase in concentration of L-tryptophan from 0.5 to 6 mg/ml, there was an increase in the production, and the maximum biosynthesis of 99.65 μg/ml IAA was achieved at 5 mg/ml L-tryptophan (Fig. 5d). IAA production decreased slightly at higher L-tryptophan concentrations. Additionally, a positive correlation between IAA production and biomass was observed.

### In vivo biocontrol of early blight disease by *Streptomyces* sp. SP5 and its impact on *Solanum lycopersicum* plant growth

**Fig. 6:**
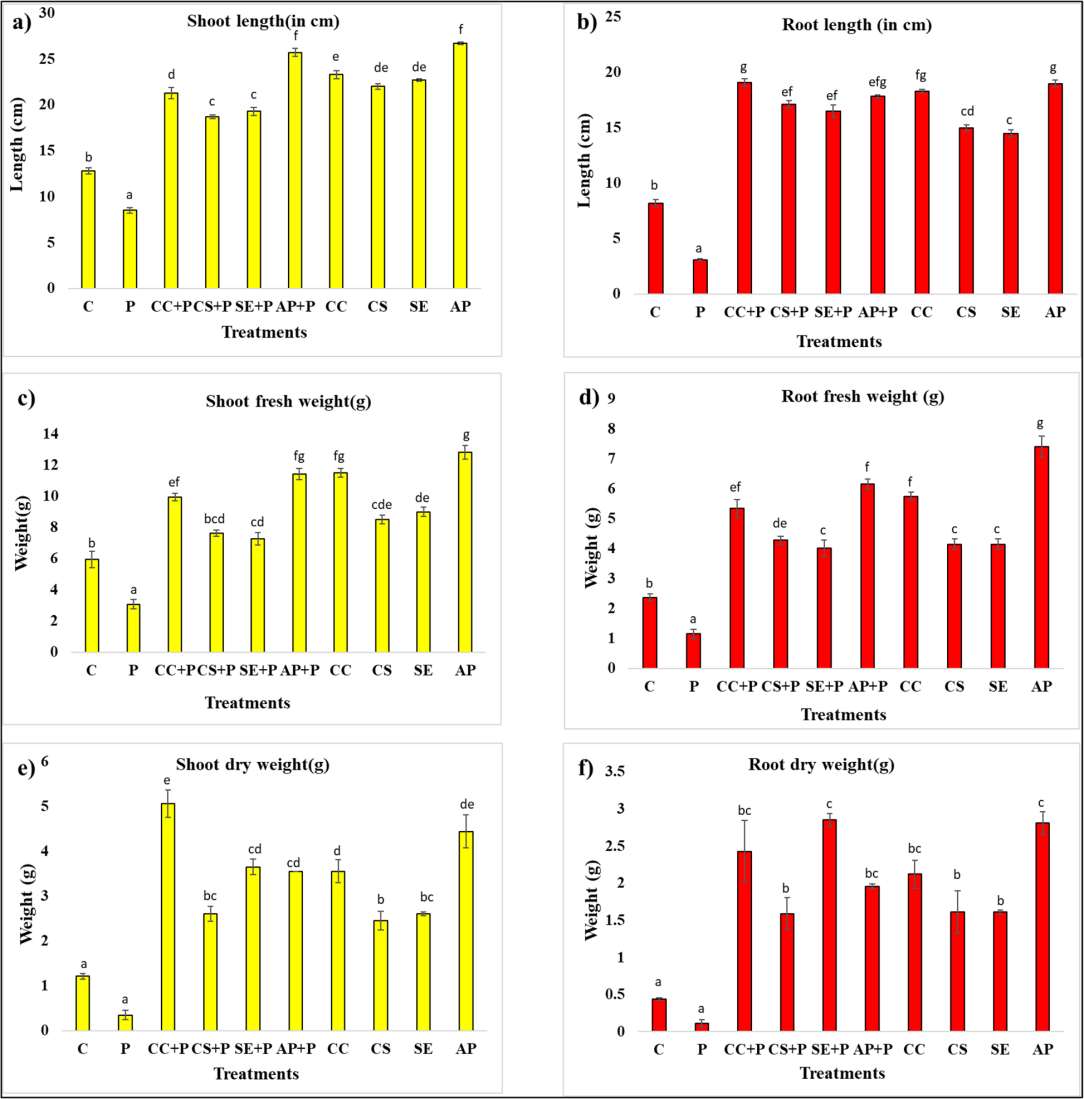
Effect of *Streptomyces* sp. SP5 and its metabolites as root treatments on *S. lycopersicum* (tomato) plants to control *Alternaria solani* causing early blight disease: **a)** shoot lengths of plants, **b**) root lengths of plants, **c)** fresh weights of shoots, **d**) fresh weights of roots, **e**) dry weights of shoots, **f**) dry weights of roots. C, control (Water only); P, pathogen only; CC + P, Streptomyces sp. SP5 cells + pathogen; CS + P, culture supernatant + pathogen; SE + P, *Streptomyces* sp. SP5 solvent extract + pathogen; AP + P, acetone precipitates + pathogen; CC, SP5 cells only; CS, culture supernatant only; SE, solvent extract only; AP, acetone precipitates only. Values were expressed in means ± standard deviation and diffrent alphabets on the graphs indicate that the average mean values of treatments are significantly different according to Tukey’s multiple comparison test (*P* ≤ 0.01)

**Fig. 7:**
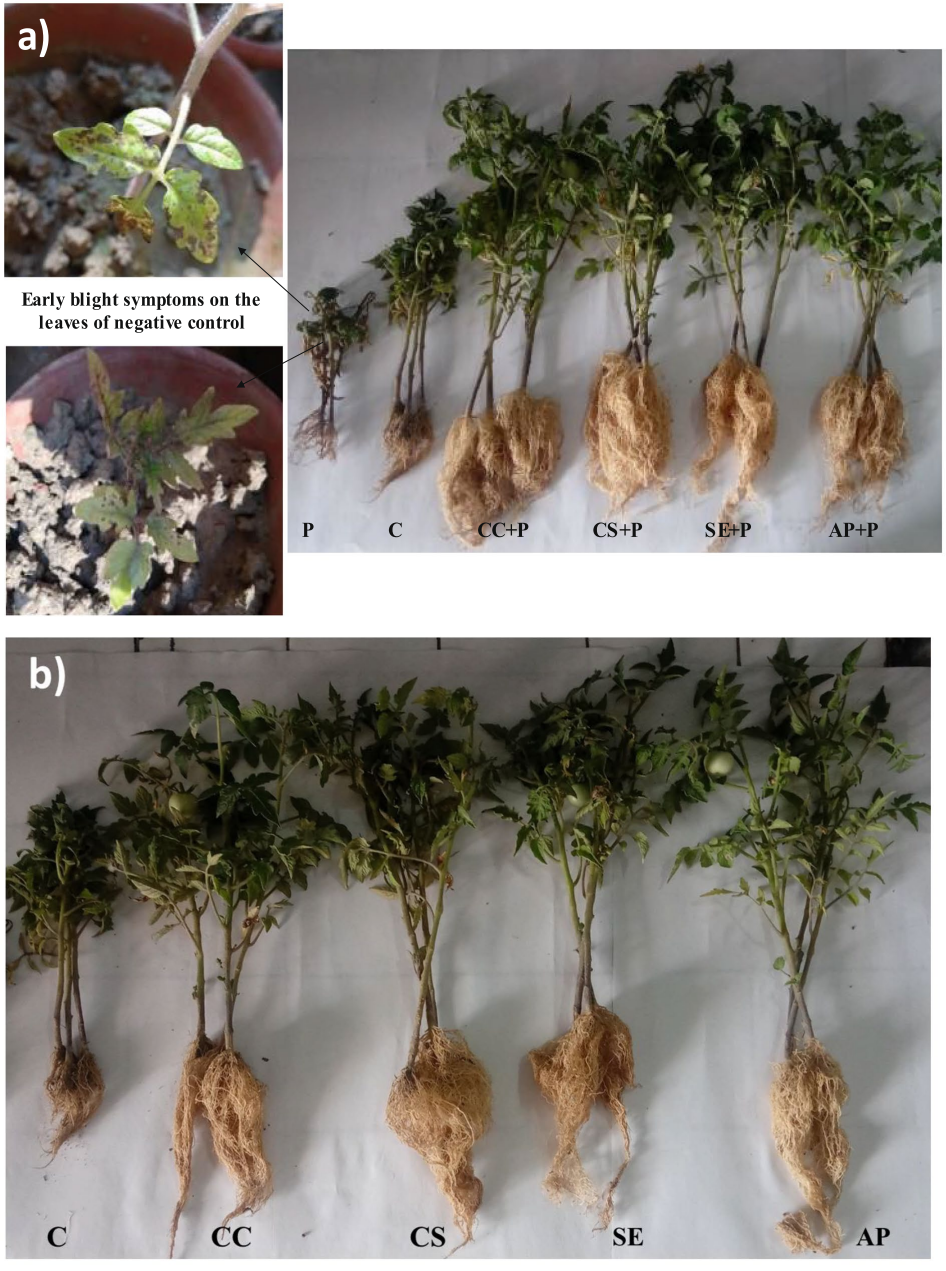
**a):** Effect of *Streptomyces* sp. SP5 and its metabolites on growth of *S. lycopersicum* (tomato) plants infected with *A. solani*; **b):** Plant growth promoting potential of *Streptomyces* sp. SP5 and its metabolites on *S. lycopersicum* (tomato) plants

Figure 6 shows the in vivo biocontrol potential of *Streptomyces* sp. SP5 against *A. solani* in *S. lycopersicum* plants. Plants treated with *Streptomyces* sp. SP5 antagonists (culture cells/culture supernatant/solvent extract/ acetone precipitates) showed no symptoms of early blight disease throughout the 3 months of experiments whereas leaf blight symptoms were observed on the negative control plants ((plants treated with pathogen only) after 1 month of experiments (Fig. 7) and at the end of experiment plants were defoliated. In addition, treatment of pathogen infected plants with *Streptomyces* sp. SP5 antagonists increased the various agronomic traits viz. shoot length (120–202.35%), root length (432–516.12%), fresh weight of shoot (135.48–270.09%), fresh weight of root (243.05–428.30%), dry weight of shoot (644.98–1346.99%) and dry weight of root (1332.4–2467.5%) significantly over the fungal pathogen infected plants (Additional file Table S6, Fig. 7a). Among the four treatments, acetone precipitates and culture cells were found to be more effective as compared to the culture supernatant and solvent extract. In the absence of pathogen stress also, *Streptomyces* sp. SP5 antagonists enhanced shoot length (71.875 to 108.54%), root length (76.82 to 131.70%), fresh weight of shoot (42.85 to 115.63%), fresh weight of root (74.15 to 264.23%), dry weight of shoot (100.98 to 264.23%) and dry weight of root (265.90 to 538.63%) over the control plants (Additional file Table S6, Fig. 7b). During the period of experimental study, early flowering and fruiting were also observed in plants treated with antagonists (culture cells/ acetone precipitates) both in the presence and absence of pathogen stress whereas no such stages were observed in control and pathogen infected plants. The *Streptomyces* sp. SP5 was re-isolated from roots, stems, and leaves of treated *S. lycopersicum* plants (Fig. S2)*.* It showed 58.3, 66.6, and 91.6% of colonization in leaves, stems, and roots, respectively (Table S7). This data indicated the biocontrol and plant growth-promoting potential of *Streptomyces* sp. SP5.

## Discussion

In this research study, a *Streptomyces* sp., designated as *Streptomyces* sp. SP5, was isolated from leaves of *Citrus jambhiri*, and characterized through a polyphasic approach. The formation of flexuous spore chains by *Streptomyces* sp. SP5 as well as chemotaxonomical markers (LL-DAP in cell wall and no characteristic sugar in whole cell hydrolysate), indicated that it belonged to the *Streptomyces* genus. In phylogenetic characterization, *Streptomyces* sp. SP5 displayed 100% sequence similarity with *Streptomyces rochei* NRRL B- 2410.

*Streptomyces* sp. SP5 inhibited varying phytopathogenic fungi (*F. moniliforme*, *A. brassicicola*, *C. herbarum, A. solani*, *F. solani*, *C. acutatum*) to varying degrees. The test pathogen may play a role in the variation in antagonistic behaviour. The genus *Streptomyces* has been studied broadly in the past decades and reported for the production of antifungal compounds [24–27]. Since the biocontrol agent's shelf-life is dependent on environmental conditions such as soil temperature, soil pH, nutrient availability and water status, one of the major issues in the commercialization of biocontrol agents is their loss of viability over time [28]. However, antifungal metabolites produced by *Streptomyces* sp. SP5 were found to be photo-thermostable and pH stable. These characteristics of metabolites make them suitable for the development of effective biocontrol agents for varied climate conditions throughout the globe.

The *Streptomyces* sp. SP5 also exhibited production of plant growth-promoting hormone, indole acetic acid (IAA). It is one of the most physiologically active auxins which stimulates and facilitates plant growth [18, 29, 30]. Biosynthesis of IAA in soil-dwelling *Streptomyces* spp. and endophytic *Streptomyces* has been documented in various studies [17, 18, 31–33]. *Streptomyces* sp. SP5 has been found to produce a significant amount (40 μg ml^− 1^) of phytohormone IAA when grown in YMB broth supplemented with tryptophan. HPLC is a reliable method for the identification of auxins, thus characterization of IAA was performed by using HPLC. Results of the classical optimization experiment showed that the incubation time, temperature, and pH played an important role in IAA production by *Streptomyces* sp. SP5 which is in agreement with other reports [34, 35]. Plant-associated microbes synthesize IAA via tryptophan (trp)-dependent and -independent pathways [36]. IAA production from *Streptomyces* sp. SP5 increased two-folds in the presence of L- tryptophan which strongly indicated that *Streptomyces* sp. SP5 synthesized IAA via the tryptophan-dependent pathway. It was also observed that beyond optimized concentrations of tryptophan, IAA production was adversely affected in *Streptomyces* sp. SP5. Myo et al. [18] also reported that high tryptophan concentrations affect the capability of strain to produce IAA. Manulis et al. [37] have reported that various *Streptomyces* spp. including *S. violaceus*, *S. griseus*, *S. scabies*, *S. coelicolor S. exfoliatus*, and *S. lividans*, produced indole- 3-acetic acid (IAA) when grown in the presence of tryptophan.

It is common knowledge that a strain's ability to suppress disease in vitro does not imply that it can be used in the fields as biocontrol agent since the strain might not be able to demonstrate its potential in natural conditions [38]. Therefore, in this study, in vivo pot experiments were conducted against the early blight of *S. lycopersicum* caused by *A. solani* to explore the possibility of using *Streptomyces* sp. SP5 as a biocontrol agent under natural conditions. Several *Streptomyces* spp. and their bioactive compounds have been reported as biocontrol agents, effective against numerous plant pathogens [9, 39, 40]. The results of the present study revealed a remarkable effect of *Streptomyces* sp. SP5 in mitigating early blight stress in *S. lycopersicum* plants. The treatment of soil, infected by *A. solani,* with *Streptomyces* sp. SP5 cells/supernatant/solvent extract and acetone precipitates exhibited a significant increase in various growth traits of *S. lycopersicum* plants and disease suppression in comparison to the plants infected with the pathogen only. To the best of our knowledge, only a few reports are available in the literature pertaining to in vivo biocontrol of early blight [11, 41–43]. The data obtained from the in vivo experimental study suggested the superiority of *Streptomyces* sp. SP5 over the earlier reported studies to control early blight of *S. lycopersicum*. Khan et al.[43] demonstrated the potential of *Paenibacillus lentimorbus* B-30488^r^ to control early blight and reported that treatment of pathogen-infected plants with *P. lentimorbus* B-30488^r^ exhibited 16% defoliation and increased the shoot length, fresh plant weight, dry plant weight and no effect on root length. On the other hand, in our study *Streptomyces* sp. SP5 completely suppressed the early blight with 0% defoliation, and no disease symptoms appeared in treated plants while complete defoliation was observed in plants exposed to the pathogen. Additionally, *Streptomyces* sp. SP5 significantly enhanced shoot length (120–202.35%), root length (432.25–516.12%), fresh weight of shoot (135.48–270.09%), fresh weight of root (243.05–428.30%), dry weight of shoot (644.98–1346.99%), and dry weight of root (1332.4–2467.5%). Similarly, Cuppels et al. [11] evaluated efficacy of strains *S. griseoviridis* K61 and *S. lydicus* WYEC108 isolated from the formulated commercial products Mycostop and Actinovate, respectively to control early blight, anthracnose and bacterial spot of field *S. lycopersicum.* Both BCAs (biocontrol agents) strongly suppressed the growth of *A. solani* in dual culture agar diffusion bioassay. *S. griseoviridis* successfully controlled anthracnose and early blight diseases whether applied before pathogen or simultaneously with the pathogen but *S. lydicus* provided protection only when applied 48 h before the exposer of pathogen.

Moreover, during the pot trial *Streptomyces* sp. SP5 also significantly increased various growth parameters of *S. lycopersicum* plants such as shoot and root lengths in the absence of pathogen stress. These findings support previous research which found that *Streptomyces* spp. not only protected plants from pathogens, but also enhanced plant growth and physiology [9, 44–46]. Dias et al. [45] observed a significant increase in shoot length and root length of *S. lycopersicum* plants treated with different *Streptomyces* isolates. However, *Streptomyces* sp. SP5 reported in the present study showed highly pronounced effect on all the observed plant growth traits in *S. lycopersicum* plants. Additionally, during this in vivo study, enhancement in some plant growth traits (root length, root fresh weight, root dry weight, shoot dry weight) was more under the pathogen stress than in the absence of pathogen stress. The reason behind these results might be the induced systemic resistance (ISR) in plants caused by pathogen stress [47, 48]. Plants possess a range of active defense apparatuses that are actively expressed in response to biotic stresses (like pathogen stress). Similarly, in our previous study we reported additional enhancement of plant growth traits (root fresh weight, shoot length) in *S. lycopersicum* pathogen-infected plants treated with *Streptomyces* sp. MR14 as compared to the plants treated with *Streptomyces* sp. MR14 alone [9].

## Conclusion

The results of in vitro and in vivo studies are highly significant and data reveals that *Streptomyces* sp. SP5 is superior to the previously reported *Streptomyces* spp. and might be used as a potential biological agent to control fungal phytopathogens and as an effective biofertilizer to promote plant growth.

## Material and methods

### Sample collection

Leaves and roots of healthy plants (*Cinnamon basil*, *Ricinus communis*, *Epipremunum aureum, Citrus jambhiri*, *Hibiscus rosasinensis*) were collected from “Botanical Garden” of Guru Nanak Dev University, Amritsar, Punjab, India (31°37′45″N and 74°49′36″N). The plants were identified and deposited in university herbarium vide accession numbers 5839, 2409, 4312, 5871 and 6462, respectively. Plant samples were wrapped aseptically in plastic bags and taken to the laboratory where they were processed within 4 hours.

### Test fungi

Different test phytopathogenic fungi viz. *Alternaria brassicicola* (MTCC2102), *Alternaria solani* (MTCC2101), *Colletotrichum acutatum* (MTCC1037), *Fusarium oxysporum* (MTCC284), *Cladosporium herbarum* (MTCC351) were procured from Microbial Type Culture Collection (MTCC), CSIR-Institute of Microbial Technology (IMTECH), Chandigarh, India. Whereas, *Fusarium solani* (NFCCI 91) was obtained from the National Fungal Culture Collection of India NFCCI, Pune. *Fusarium* sp. and *Alternaria* sp. (accession number GU004283) were isolated in the lab [49]. All the fungal cultures were maintained at 4 °C on Potato dextrose agar (PDA) slants.

### Isolation of endophytic strains from plant samples

The collected plant samples were washed under running tap water for 1–2 minutes to remove soil particles. The samples were then surface sterilized by 70% ethanol for 10 min followed by treatment with 1% sodium hypochlorite solution for 15 min [50]. Plant samples were then repeatedly washed with sterilized water, air-dried in a laminar airflow hood, and cut into small pieces using a sterile razor blade. The small pieces of sterile samples were placed on SCNA medium (starch casein nitrate agar). To inhibit the growth of fungi and Gram negative bacteria, cycloheximide (50 g/ml) and nalidixic acid (50 g/ml) were added to the medium. The plates were then incubated at 28 °C for 7–21 days. Isolated actinobacteria colonies were subcultured and purified on SCNA plates. Isolate spores were reserved as a stock in 20% glycerol at − 20 °C for potential use.

### Screening and selection of the isolate

Primary screening determines the ability of the microorganisms to produce an antifungal metabolite without providing a significant idea about the production potential of the organism. Modified Kirby Bauer antibiotic susceptibility test was used for primary screening [51]. The actinobacterial isolates were cultured for 7 days on the SCNA medium at 28 °C. Six mm agar discs from well-grown cultures of actinobacterial isolates were placed on PDA plates which were already seeded with the test phytopathogenic fungi (100 μl of 10^6^spores ml^− 1^). The plates were then kept at 4 °C for 30 min for the diffusion and then incubated at 28 °C. The zones of inhibition were determined after 48 h.

Following primary screening, the active isolates were subjected to secondary screening [52]. Three discs of actinobacterial culture with a diameter of 6 mm were transferred to 250 ml Erlenmeyer flasks containing 50 ml of starch casein nitrate broth and incubated for 7 days at 28 °C in a rotary shaker at 180 rpm. Mycelia were removed by centrifuging at 10,000 x g for 20 minutes at 4 °C and supernatant was used for bioassay. The PDA plates inoculated with test fungi (100 μl of 10^6^spores ml^− 1^) were punctured with sterile cork borer to create wells (6 mm in diameter) and 200 μl of culture supernatant was transferred to each well under aseptic conditions. The plates were incubated at 28 °C for 3 days and observed for antifungal activity of the isolates as clear zones of inhibition around wells. Out of 9 isolates from secondary screening, isolate SP5 was selected for further studies based on its broad-spectrum antifungal activity.

### Morphological, physiological, and biochemical characterization of *Streptomyces* SP. SP5

Cultural characteristics were determined as per methods described by Shirling and Gottlieb [53] in the International *Streptomyces* Project (ISP). ISCC-NBS standard color chart was used to determine the colors of the substrate and aerial mycelia [54]. Brightfield light microscopy and electron microscopy were used to examine the isolate morphological characteristics. Physiological and biochemical characterization such as growth at different temperatures (20–50 °C), pH (5.0–12.0), salt concentration (0–20% w/v), and capacity to generate different hydrolytic enzymes were carried out according to standard protocols [55]. The assimilation of sugars as carbon source was investigated as per Shirling and Gottlieb [53]. The major diagnostic features of *Streptomyces*, sugar pattern in whole-cell hydrolysate and isomer of diaminopimelic acid (DAP) in the cell wall, were determined using Lechevalier and Lechevalier [56].

#### Genomic characterization

The genomic DNA of the isolate was extracted using Marmur method [57]. The 16S rRNA gene sequence was amplified by polymerase chain reaction (PCR) using primers 27f (5′-AGAGTTTGATCC TGGCTCAG-3′) and 1492r (5′-AGAAAGGAGGTGATC CAGGC-3′). Amplification was done for 40 cycles in a PCR machine (Eppendorf-gradient). Each cycle included a denaturation stage of 1 minute at 94 °C, an annealing step of 1 minute at 50 °C, an extension step of 2 minutes at 72 °C and a final extension of 10 minutes at 72 °C. The QIA quick gel extraction kit (Qiagen, Germany) was used to purify the obtained PCR product. The Institute of Microbial Technology (IMTECH), Chandigarh, India, sequenced the 16S rRNA gene of *Streptomyces* sp. SP5. For the detection of phylogenetic neighbors and calculation of pairwise 16S rRNA gene sequence similarities, the EzTaxon server (http://www.ezbiocloud.net) was used [58]. The Clustal W program was used to align the nearly complete sequence (1400 bp). Phylogenetic tree based on bootstrap values (1000 replications with MEGAX software) was constructed using neighbor-joining method [59, 60].

#### Time course experiment related to antifungal metabolite production and growth

For antifungal profiling, seed culture was prepared in Erlenmeyer flask (250 ml) containing 50 ml of starch casein nitrate broth, inoculated with three discs (6 mm diameter) of 7 days old culture grown on SCNA and incubated at 28 °C for 3 days at 180 rpm. Production was carried out by inoculating the production medium (SCN broth) with 2% seed culture (10^6^ colony forming units /ml)) and incubating at 28 °C for 10 days of agitation at 180 rpm. After every 24 h, the flasks were harvested and culture broth was centrifuged at 10,000×g for 10 min. The biomass was separated from the culture supernatant and dried at 60 °C for 2 days. The activity against test fungal phytopathogens was determined using the remaining cell-free culture supernatant. The test fungi (100 μl of 10^6^spores ml^− 1^) were seeded on PDA plates, which were punctured with sterile cork borer to create 6 mm wells. The plates were held in the refrigerator for 1 hour after adding 200 μl of culture supernatant to allow active metabolites to diffuse. In the control wells, culture supernatant was replaced with water/uninoculated broth. After diffusion, the plates were incubated at 28 °C for 2–3 days. The antifungal activity of the isolate was measured in millimetres as zones of inhibition around wells.

#### Recovery of antifungal metabolites

For the production of antifungal active metabolites, fermentation was carried out by inoculating the culture (at a concentration of 2.5% from seed culture) in production medium and incubating at 28 °C under shaking (180 rpm). Fermentation was terminated when the maximum antifungal activity was observed (5th day) and culture broth was centrifuged at 10,000×g at 4 °C for 10 min to separate the mycelium.

The active metabolites from culture supernatant were recovered by solvent extraction. Different organic solvents (hexane, diethyl ether, chloroform, ethyl acetate, butanol) having a wide range of polarity were screened to work out the best extractant. The supernatant of *Streptomyces* sp. SP5 was extracted twice in the ratio of 1:1 (supernatant: solvent). Rotavapor (BUCHI Rotavapor R-210) was used to concentrate the obtained organic phase to dryness. The obtained crude extracts were redissolved in respective solvents (1 ml) and tested for antifungal activity against test fungi. Acetone precipitation was also employed to extract out the antifungal metabolites from the culture supernatant. For acetone precipitation, two volumes of 100% chilled acetone were added to the culture supernatant and kept for 1 hour at -20 °C. The precipitates were recovered by centrifugation at 10,000×g for 10 minutes at 4 °C, and were redissolved in sterile distilled water. The antifungal activity of precipitates was determined against different test phytopathogenic fungi using the agar well diffusion method.

#### Stability of active metabolites in the culture supernatant

The supernatant was heated at various temperatures (37 °C, 50 °C, 70 °C, 100 °C and autoclaving at 121 °C) for 1 hr, and was also exposed to a lower temperature (− 20 °C) for 1 hour to determine thermostability. The effect of pH on activity was evaluated by adjusting the pH at 2.0 and 14.0, incubating for 1 hr at 28 °C, and then reverting the pH of the supernatant to 7.0 to check antifungal activity. Photostability was tested by exposing the supernatant separately to UV (200 – 600 nm) and sunlight (1 hr). The antifungal activity of all the treated samples was then tested using well diffusion assay and values were represented as their mean ± SD of three independent experiments.

### Plant growth promoting potential

#### Indole acetic acid (IAA) production

For Indole acetic acid (IAA) production, 50 ml of yeast malt broth (YMB (Himedia); g/l: yeast extract 4.0, malt extract 10.0, dextrose 4.0; pH 6.8 ± 0.2) containing 0.2% L-tryptophan was inoculated with three plugs (6 mm) of seven-days-old *Streptomyces* sp. SP5 culture grown on SCNA and then incubated at 28 °C for 7 days under shaking (180 rpm). After incubation, the broth was centrifuged at 10,000x g for 15 minutes to remove mycelia. To detect IAA production, 2 ml Salkowski reagent was added into tubes containing 1 ml of cell-free supernatant and incubated for 25 min at room temperature. The intensity of developed pink color was measured by taking absorbance at 530 nm using Shimadzu UV-visible spectrophotometer (UV-1601, Japan), which indicates indole acetic acid production. The concentration of IAA produced was estimated against the standard curve of IAA using a concentration range of 10–100 μg/ml.

#### Extraction, purification, and identification of indole acetic acid

The culture supernatant was acidified to pH 2.5 with HCl and extracted twice with ethyl acetate using solvent extraction. The separated organic phase was concentrated using a rotary evaporator and redissolved in ethyl acetate. For IAA analysis, the ethyl acetate extract was separated using thin-layer chromatography (TLC) with propanol: water (8:2, v/v) as solvent system, and the chromatogram was observed after spraying the plates with Salkowski regent. IAA was partially purified from the crude solvent extract by using silica gel column chromatography and fractions were collected with the solvent system of ethyl acetate and hexane (20:80 v/v). Each fraction was checked for the presence of IAA by TLC using Salkowski regent. The indole containing fraction was further quantified using reversed-phase high performance liquid chromatography (RP-HPLC): Shimadzu Micros orb MV, 100 mm × 10 mm ID, 10 μm, using C-18 reversed-phase column with methanol: 1% acetic acid in water (40:60 v/v as mobile phase) as an eluent at 1 ml min^− 1^ flow rate. For the analysis, 20 μl of partially purified IAA extract was spiked into the column and pure IAA compound was used as a standard. The identical retention time of the respective standard was used to assess the presence of IAA in the purified fraction.

#### Optimization of IAA production

To optimize IAA production, various factors such as incubation period, pH, temperature, and tryptophan concentration were studied using one variable at a time approach. In order to determine the effect of incubation time, 50 ml of YMB was added in each of 250 ml Erlenmeyer flasks which were then inoculated with 2% v/v inoculum (10^6^ cfu/ml, developed as mentioned above in the time course experiment) and incubated at 28 °C at 180 rpm for 10 days. The effect of pH on IAA production was determined by changing the initial pH of the medium from 2 to 10 using 1 N HCl and 1 N NaOH. Similarly, the effect temperature of incubation on production was investigated by incubating *Streptomyces* sp. SP5 into the same medium at 20, 25, 28, 30, 35, and 40 °C for 6 days (at which the maximum production was achieved). The effect of L-tryptophan concentration was determined by varying L-tryptophan over a range of 1–5 mg/ml in the medium (pH 7.0). All experiments were performed in triplicates and average values were observed.

#### In vivo biocontrol of *A. solani* by *Streptomyces* SP. SP5 and its effect on *S. lycopersicum* plant growth

Efficacy of *Streptomyces* sp. SP5 against early blight disease was tested by conducting three independent pot trials at Guru Nanak Dev University (Amritsar, Punjab, India) using soil drenching method. The aim was to observe whether *Streptomyces* sp. SP5 culture cells, culture supernatant, solvent extract as well as acetone precipitates could regulate early blight caused by *A. solani* and promote various plant growth traits. *S. lycopersicum* seeds susceptible to *A. solani* (*S. lycopersicum* Mill. “Pusa Ruby”) were sown in sterile soil for 3 weeks at 28 ± 2.0 °C and after true leaf stage, a single plant was transplanted into a pot of 8 cm diameter containing 100 g sterile soil for each treatment. The plants were subjected to a variety of treatments with *Streptomyces* sp. SP5 antagonists viz. *Streptomyces* sp. SP5 cells, supernatant, solvent extract and acetone precipitates, with and without pathogen (Table 2). Three replications were maintained for each treatment and the pots were kept under natural conditions (temperature 22 ± 2.0 °C, photoperiod L16: D8). Plants were watered daily and observed for disease symptoms, flowering, and fruiting stages. After disease appearance in the control plants, all plants were uprooted carefully, and washed with running tap water to remove the adhered soil. The shoot & root lengths and fresh & dry weights of seedlings were recorded, and data obtained were subjected to statistical analysis. Values were represented as mean ± SD of three independent experiments, and to compare the mean difference, one-way analysis of variance (ANOVA) with Tukey’s post hoc test was carried out using SPSS statistical analysis software (Version 20.0, IBM SPSS). To determine the endophytic relationship, *Streptomyces* sp. SP5 was re-isolated from different parts (roots, stems, and leaves) of treated *S. lycopersicum* plants*.* Culture obtained from plant parts was identified by morphological, physiological, and biochemical characterization. The recovery of colony growth of *Streptomyces* sp. SP5 on the explant pieces was evaluated and calculated for colonization percentage using the following formula:

1 $$\textrm{Colonization}\left(\%\right)=\frac{\textrm{Number}\kern0.17em \textrm{of}\kern0.17em \textrm{plant pieces}\kern0.17em \textrm{with}\kern0.17em \textrm{colony}\kern0.17em \textrm{growth}}{\textrm{Total}\kern0.17em \textrm{number}\kern0.17em \textrm{of}\kern0.17em \textrm{plant}\kern0.17em \textrm{pieces}}\times 100$$

**Table 2:** Treatment of *S. lycopersicum* plants with *Streptomyces* sp. SP5 antagonists by soil drenching method

Group Name	Treatments
**P** (Fungal pathogen)	10 ml of fungal spore suspension (1 × 10^6^ spores/ ml)
**C** (Control)	water only
**CC** + **P** (Culture cells and pathogen)	10 ml of fungal spore suspension (1 × 10^6^ spores/ml) and 10 ml of *Streptomyces* sp. SP5 culture cell suspension (1 × 10^6^ cells/ml)
**CS** + **P** (Culture supernatant and pathogen)	10 ml of fungal spore suspension (1 × 10^6^ spores/ml) and 10 ml of culture supernatant of *Streptomyces* sp. SP5
**SE** + **P** (Solvent extract and pathogen)	10 ml of fungal spore suspension (1 × 10^6^ spores/ml) and 10 ml of solvent extract (1 mg/ml)
**AP** + **P** (Acetone precipitates and pathogen)	10 ml fungal spore suspension (1 × 10^6^ spores/ml) And 10 ml of culture precipitates dissolved in water (250 μg/ml)
**CC** (Culture cells)	10 ml of *Streptomyces* sp. SP5 culture cell suspension (1 × 10^6^ cells/ml)
**CS** (Culture supernatant)	10 ml of culture supernatant of *Streptomyces* sp. SP5
**SE** (Solvent extract)	10 ml of solvent extract of *Streptomyces* sp. SP5 (1 mg/ml)
**AP** (Acetone precipitate)	10 ml acetone precipitate (250 μg/ml)

## Supplementary Information

**Additional file 1.**

## Data Availability

All data generated or analysed during this study are included in this article.

## Abbreviations

SCNA: Starch Casein Nitrate Agar
ISP: International *Streptomyces* Project
TLC: Thin Layer Chromatography
PDA: Potato Dextrose Agar
DAP: Diaminopimelic Acid
MTCC: Microbial Type Culture Collection
IAA: Indole Acetic Acid
