# Supplementary material for: Investigating the plant growth promoting and biocontrol potentiality of endophytic Streptomyces SP. SP5 against early blight in Solanum lycopersicum seedlings
Source: BMC Microbiol. 2022 Nov 29;22:285. doi: 10.1186/s12866-022-02695-8 (PMC9706909; doi:10.1186/s12866-022-02695-8)
Supplement: Supplementary file 1 — Additional file 1. [file 12866_2022_2695_MOESM1_ESM.docx]

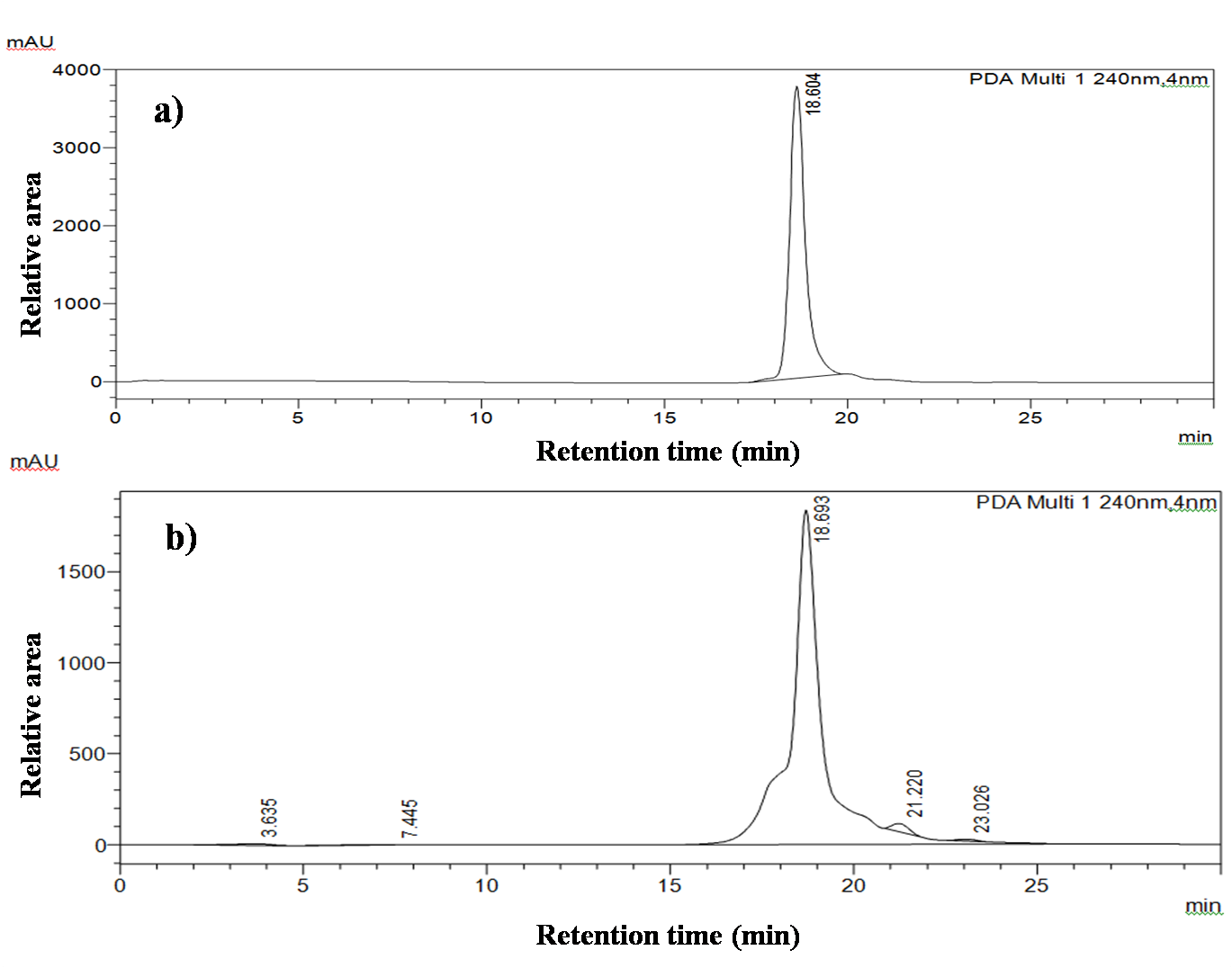


**Fig. S1** Semi preparative HPLC chromatogram of a) IAA standard and b) partial purified extract of culture filtrate of *Streptomyces* sp. SP5


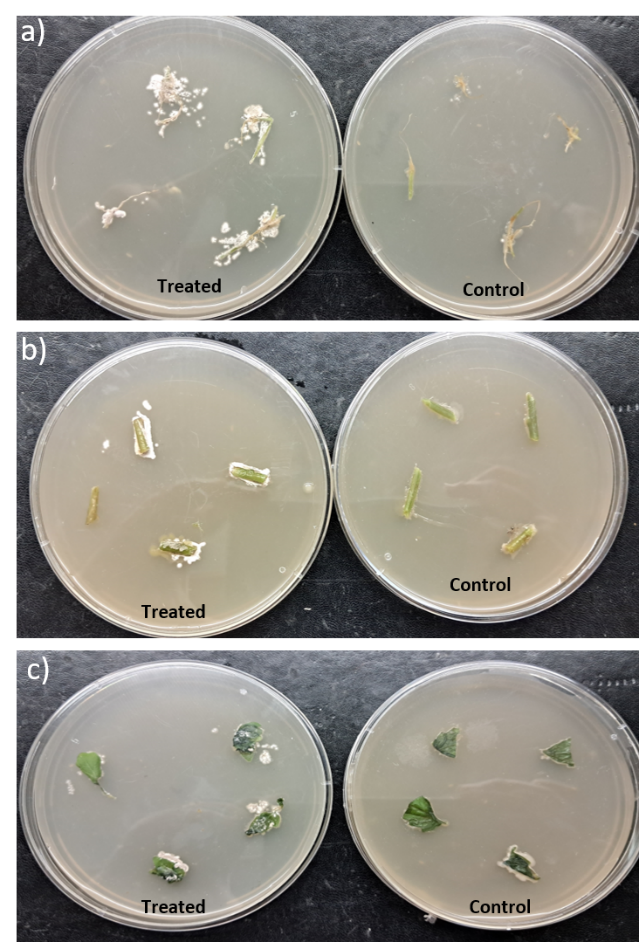


**Fig.S2** *Streptomyces* sp. SP5 reisolation: a) isolation from roots; b) isolation from stem; c) isolation from leaves (Treated: plants treated with SP5 cells and pathogen; Control: water only)

**Table S1: *In vitro* antifungal activity of cell-free culture supernatant of active isolates against various fungal phytopathogens**

| **Isolate** | ***A. brassicicola*** | ***C. herbarum*** | ***F. moniliforme*** | ***F. oxysporum*** | ***A. solani*** | ***F. solani*** |
| --- | --- | --- | --- | --- | --- | --- |
|  | **Zone of inhibition (mm)** | | | | | |
| **SP1** | 13.5 ± 0.5 | - | - | 10.83 ± 0.29 | - | 10.83 ± 0.76 |
| **RC8** | 14.17 ± 0.29 | - | - | - | - | 10.33 ± 0.29 |
| **RS29** | 19.67 ± 0.58 | 17.17± 0.76 | - | - | 16.17 ± 1.04 | 10.33 ± 0.58 |
| **HS9** | 15.33 ± 0.58 | 10.17± 1.04 | - | 13.5 ± 1.5 | - | - |
| **EA10** | 17.33 ± 0.76 | - | - | 10.5 ± 0.5 | 13.17 ± 1.04 | 19.33 ± 1.15 |
| **SP5** | **22.0 ± 0.5** | **22.33± 1.15** | **18.0 ± 0.0** | **20.67 ± 0.58** | **23.0 ± 1.0** | **17.17 ± 0.29** |
| **E21** | 16.33 ± 0.58 | - | - | - | - | 12.5 ± 0.5 |
| **RS5** | 17.83 ± 0.29 | - | - | 14.5 ± 0.5 | 15.17 ± 0.29 | - |
| **E17** | 13.83 ± 0.76 | - | 16.5 ± 0.5 | - | 10.75 ± 0.5 | - 1. 0.76 |

**Values represented as mean ± SD (n = 3); - means no zone of inhibition**

**Table S2 Cultural characteristics of *Streptomyces* sp. SP5 on SCNA and various ISP media**

| **Medium** | **Spore mass color** | **Substrate**  **mycelium** | **Aerial**  **mycelium** | **Growth** | **Diffusible pigment** |
| --- | --- | --- | --- | --- | --- |
| **SCNA** | Yellowish white | Brilliant yellow | Strong yellow | Good | _ |
| **ISP1** | Yellowish white | Brilliant yellow | Strong yellow | Good | _ |
| **ISP2** | - | Yellowish white | Yellowish white | Poor | _ |
| **ISP3** | Moderate yellowish brown | Strong yellowish brown | Deep yellowish brown | Good | _ |
| **ISP4** | Deep yellowish brown | Moderate olive brown | Light olive brown | Good | _ |
| **ISP5** | Yellowish white | Light olive brown | Light olive brown | Good | _ |
| **ISP6** | Yellowish grey | Strong yellowish brown | Dark yellow | Good | _ |
| **ISP7** | Yellowish white | Dark orange yellow | Strong yellowish brown | Good | _ |

**Table S3 Characteristics of *Streptomyces* sp. SP5**

| **Characteristic** |  |
| --- | --- |
| **Morphological characteristics**Spore chain  Spore shape  Spore surface | Spiral  RoundSmooth |
| **Chemotaxonomical markers**  Sugar pattern  Diaminopimelic acid | No characteristic sugar  LL-DAP |
| **Physiological characteristics**  Salt tolerance  Temperature tolerance  pH tolerance | 5.0%  20°C to 45°C  5-11 |
| **Production of Melanoid pigment**  Tyrosine agar medium (ISP 7)  Peptone Yeast extract agar medium | -  - |
| **Biochemical characteristics**  Indole production  Methyl red test  Voges-Proskauer test  Citrate utilization  Cellulose hydrolysis  Casein hydrolysis  Catalase activity  Urea hydrolysis  Esculin hydrolysis  Starch hydrolysis  Lipid hydrolysis  Gelatin hydrolysis  Hydrogen sulphide production  Oxidase test  **Utilization of sugars**  Lactose  D-Glucose  Sucrose  Lactose  Inositol  Xylose  Fructose  Arabinose  Rhamnose  Raffinose | -  -  -  +  +  -  +  +  +  +  +  +  +  +  +  +  +  +  +  +  +  +  -  +  + |

**Table S4 Activity profile of *Streptomyces* sp. SP5 against fungal pathogens.**

| **Incubation**  **Day** | **Zone of inhibition (mm)**  **Test fungi** | | | | | **Biomass**  **(mg/50 mL)** |
| --- | --- | --- | --- | --- | --- | --- |
|  | ***F. oxysporum*** | ***C. herbarum*** | ***F. moniliforme*** | ***A. solani*** | ***A. brassicicola*** |  |
| **1** | - | - | - | - | - | 28±1 |
| **2** | 15.66±0.6 | 14.33±1.2 | 17.33±0.9 | 14.66±0.6 | - | 34±1.1 |
| **3** | 15.66±1.2 | 16.66±0.6 | 18.66±1.2 | 19.66±1.5 | 15±0.9 | 36±3.4 |
| **4** | 18.33±1.2 | 19±1.1 | 19±0.9 | 20±1.7 | 19±0.9 | 74.33±1.1 |
| **5** | **25.33±0.6** | **20.33±1.3** | **22.67±1.2** | **24±1.7** | **21±0.9** | **146±2.4** |
| **6** | 14±1 | 17.33±0.7 | 15.66±0.6 | 14.66±0.7 | 19±0.9 | 133±1.9 |
| **7** | 9.66±0.6 | 11±0.7 | - | 9.33±0.5 | 11±0.5 | 107±1.8 |
| **8** | - | 9.66±0.5 | - | - | 9±1.2 | 106±2.1 |
| **9** | - | - | - | - | - | 85±3.6 |
| **10** | - | - | - | - | - | 84.67± 2.1 |

**Values represented as mean ± SD (n = 3); - means no zone of inhibition**

**Table S5 Antifungal activity of culture supernatant of *Streptomyces* sp. SP5**

| **Test fungi** | **Zone of inhibition (mm)** |
| --- | --- |
| *F. oxysporum* | 26±0.6 |
| *C. herbarum* | 25±0.8 |
| *F. moniliforme* | 28±0.6 |
| *A. brassicicola* | 22±0.9 |
| *C. acutatum* | 23±0.6 |
| *A. solani* | 20±1.1 |
| *A. alternata* | 24±0.7 |
| *F. solani* | 22±1.4 |

**Values represented as mean ± SD (n = 3)**

**Table S6 Biocontrol and plant growth promoting effect of *Streptomyces* sp. SP5 on various growth traits of *S. lycopersicum* seedlings infected with fungal phytopathogen *A. solani***

| **Treatment** | **Shoot length**  **(cm)** | **Root Length**  **(cm)** | **Shoot fresh weight (g)** | **Root fresh weight (g)** | **Shoot dry weight**  **(g)** | **Root dry weight**  **(g)** |
| --- | --- | --- | --- | --- | --- | --- |
| **C** | 12.8±0.57 | 8.2±0.57 | 5.95±0.91 | 2.38±0.20 | 1.219±0.11 | 0.44±0.02 |
| **P** | 8.5±0.3 | 3.1±0.15 | 3.08±0.52 | 1.16±0.28 | 0.349±0.18 | 0.111±0.08 |
| **CC+P** | 21.3±1.04 (150.58) ^*^ | 19.1±0.57  (516.12) * | 9.95±0.42  (222.73) * | 5.34±0.53  (357.97) * | 5.05±0.10  (1346.99) * | 2.43±0.72  (2109.09) * |
| **CS+P** | 18.7±0.40  (120) * | 17.1±0.57  (451.61) * | 7.61±0.34  (146.83) * | 4.28±0.25  (267.06) * | 2.60±0.28  (644.98) * | 1.59±0.36  (1332.4) * |
| **SE+P** | 19.3±0.76  (127.05) * | 16.5±0.1  (432.25) * | 7.26±0.67  (135.48) * | 4.00±0.48  (243.05) * | 3.64±0.30  (945.27) * | 2.85±0.14  (2467.5) * |
| **AP+P** | 25.7±0.76  (202.35) * | 17.83±0.28  (475.16) * | 11.41±0.62  (270.09) * | 6.16±0.28  (428.30) * | 3.55±0.43  (917.76) * | 1.96±0.06  (1666.66) * |
| **CC** | 23.3±0.76  (82.03) ** | 18.3±0.28  (123.17) ** | 11.50±0.5  (93.27) ** | 5.75±0.2  (141.29) ** | 3.55±0.43  (191.22) ** | 2.12±0.32  (381.81) ** |
| **CS** | 22±0.0.5  (71.875) ** | 15±0.5  (82.92) ** | 8.50±0.5  (42.85) ** | 4.15±0.30  (74.15) ** | 2.45±0.36  (100.98) ** | 1.61±0.49  (265.90) ** |
| **SE** | 22.7±0.28  (77.34) ** | 14.5±0.5  (76.82) ** | 9±0.5  (51.26) ** | 4.15±0.29  (74.15) ** | 2.602±0.08  (113.45) ** | 1.61±0.05  (265.90) ** |
| **AP** | 26.7±0.25  (108.59) ** | 19±0.5  (131.70) ** | 12.83±0.76  (115.63) ** | 7.41±0.62  (210.95) ** | 4.44±0.63  (264.23) ** | 2.81±0.26  (538.63) ** |

All data are presented as mean ± SD (n=3) of three independent experiments. C = Control, P = Pathogen, CC+P = Culture cells and pathogen, CS+P = Culture supernatant and pathogen SE+P = Solvent extract and pathogen, AP+P = Acetone precipitate and pathogen, CC = Culture cells, CS = Culture supernatant, SE = Solvent extract, AP = Acetone precipitate. * Values indicate percentage increase over pathogen infested plants and ** values indicate percentage increase over control plants **(Tukey’s Test P≤0.01).**

**Table S7: Colonization percentage of *Streptomyces* sp. SP5 in *S. lycopersicum* plant parts.**

| **Colonization percentage (%)** | ***S. lycopersicum* plant parts** | | |
| --- | --- | --- | --- |
|  | **Root** | **Stem** | **Leaves** |
|  | 91.6 | 66.6 | 58.3 |
